# The impact of swithching faricimab in the treatment of neovascular age-related macular degeneration: a real-world analysis

**DOI:** 10.1007/s00417-025-06931-5

**Published:** 2025-08-18

**Authors:** Giacomo Boscia, Devinder Chauhan, Giulia Corradetti, Charles P. O’Neill, Gihan Samarasinghe, Sophiana M. Lindenberg, Anna Urrea, Stephanie Mauger, Giang Do, Muneeswar Gupta, SriniVas R. Sadda

**Affiliations:** 1 Doheny Eye Institute, 150 N Orange Grove Blvd, Pasadena, CA USA; 2 Department of Translational Biomedicine Neuroscience, University of Bari “Aldo Moro”, Bari, Italy; 3 Macuject Pty Ltd, Melbourne, VIC Australia; 4 Department of Ophthalmology, David Geffen School of Medicine at UCLA, Los Angeles, CA USA; 5 Australian National University, Canberra, ACT Australia

**Keywords:** Neovascular age-related macular degeneration, Anti-VEGF therapy, Faricimab, Subretinal fluid, Intraretinal fluid

## Abstract

**Background:**

To report a real-world analysis of switching to faricimab in the setting of neovascular age-related macular degeneration (nAMD), with regards to fluid volumes on optical coherence tomography (OCT), treatment interval, and best corrected visual acuity (BCVA).

**Methods:**

We retrospectively collected the OCT scans of all eyes that were being treated with anti-vascular endothelial growth factor (VEGF) therapy and were switched to faricimab in clinical the practices of eight retina specialists at five clinics in three Australian states (CORRNet Study Group; Clinical Ophthalmologists’ Real-world Research Network). Only those that remained on faricimab are the subject of this report. All OCT scans were analyzed for both the presence and volume of intraretinal fluid (IRF) and subretinal fluid (SRF) using machine learning (ML) algorithms (Macuject Pty Ltd, Melbourne, Australia). Mean interval time between injections and BCVA were recorded at the time of switch and after the switch to faricimab.

**Results:**

Of the 3082 eyes of 2200 patients treated with intravitreal anti-VEGF injections for nAMD, 473 eyes (84.0% of those switched to faricimab) remained on faricimab and thus met the criteria to be included in the analysis. At the time of switch to faricimab the number of eyes having IRF, either alone or in combination with SRF, was 142 (30.0%) and the number of eyes with SRF, either alone or in combination with IRF, was 223 (47.2%).

Following the switch to faricimab, for the 142 eyes with any IRF, 115 eyes (80.9%) had a reduction in IRF volume, and of the 223 eyes with SRF, 190 eyes (85.2%) had a reduction in SRF volume, and 64 of the 69 (92.8%) eyes with both IRF and SRF present had a reduction in both fluid subtypes. The interval distribution shifted from a mean of 5.78 ± 1.92 (SD) to 6.91 ± 2.26 (SD) weeks over the study period (*p* < 0.001). The mean BCVA improved from 63.91 ± 20.03 logMar letters to 69.25 ± 17.38 logMar letters (*p* < 0.001) after the switch.

**Conclusions:**

Switching to faricimab in eyes with nAMD previously treated with other anti-VEGF agents was associated with anatomical and functional improvements in this selected cohort. Further prospective studies including the comparison with other anti-VEGF agents are warranted to more definitively evaluate the clinical benefits of faricimab in this setting.

## Abstract (highlights)

**Key messages:**

*****What is known***:**

- Neovascular age-related macular degeneration (nAMD) is a leading cause of vision loss, and anti-VEGF therapies have significantly improved patient outcomes, although treatment burden remains high due to the need for frequent injections.
- Faricimab, a bispecific antibody targeting VEGF-A and Ang2, has demonstrated non-inferior efficacy in clinical trials compared to aflibercept, with the potential for extended treatment intervals.

*****What is new***:**

- This real-world study provides machine-learning-based volumetric analysis of intraretinal and subretinal fluid dynamics in patients switched from other anti-VEGF agents to faricimab, demonstrating significant reductions in fluid volume and extended fluid-free treatment intervals.
- Unlike previous studies, this analysis reveals a significant improvement in best corrected visual acuity (BCVA) following the switch to faricimab, suggesting a potential benefit in functional outcomes for “hard-to-treat” nAMD patients.

## Introduction

Age-related macular degeneration (AMD) is a leading cause of vision loss affecting over 6 million people globally [1]. The neovascular form of AMD (nAMD), also known as exudative or wet AMD, results in severe vision loss due to the development of exudative macular neovascularization (MNV), commonly extending from the choriocapillaris and into the sub-RPE or subretinal space (Type 1 and 2 MNV, respectively) [2, 3]. The exudation and accumulation of intraretinal and subretinal fluid (IRF and SRF) from MNV, is associated with dysfunction and loss of retinal photoreceptors and retinal pigment epithelium (RPE) cells over time, leading to visual impairment [3].

The introduction of anti-vascular endothelial growth factor (anti-VEGF) therapy has revolutionized the management of nAMD [4], with several intravitreal anti-VEGF agents now available in routine clinical practice. Nevertheless, given the extensive treatment burden of ongoing, often lifelong, intravitreal injections [5], treating clinicians have adopted modifications of treatment regimens such as “treat and extend regimen” (TER) [6], and newer agents have been developed to control fluid exudation better and last longer [7–9].

In 2022, the Food and Drug Administration (FDA) approved faricimab (Vabysmo, RG7716, Roche/Genentech, Basel, Switzerland), which is a bispecific antibody targeting both VEGF-A and angiopoietin2 (Ang2) [10]. Ang2 is a component of the Ang/Tie pathway playing a crucial role in vascular homeostasis by influencing vascular permeability and angiogenesis [11]. Consequently, the bispecific blockage of both these two proangiogenic factors by faricimab was hypothesized to result in stronger inhibition of exudation and increased vascular stability [12].

The results of the two multi-center, randomised, double-masked, non-inferiority Phase 3 trials, TENAYA and LUCERNE, conducted across 271 sites worldwide, showed that in 1329 treatment naïve patients with nAMD, those receiving faricimab 6.0 mg at an interval of up to 16 weeks obtained non-inferior functional and anatomical results compared to those receiving intravitreal aflibercept 2.0 mg (Eylea/Regeneron; NY, USA) at an 8 week interval after 48 weeks of follow up [10, 12]. In the TENAYA and LUCERNE studies, faricimab demonstrated a higher rate of fluid resolution compared to aflibercept during the initial loading phase, underscoring its potential for both short- and long-term disease control [10].

However, it is also important to report the outcomes of these agents in real-world clinical practice, particularly for those patients in which complete fluid resolution is difficult to achieve, or the interval between injections is shorter than desired. Unsurprisingly, the earliest real-world reports on faricimab utilization studied nAMD eyes that were switched from one of the previously available anti-VEGF agents to faricimab [13–15].

Whilst the measurement of treatment interval is simple, measurement of fluid volume is not. Indeed, although fluid accumulates in a three-dimensional compartments within (IRF) or under (SRF) the neurosensory retina, measurement in clinical trials and practice is usually simply in one dimension - macular thickness, or central subfield thickness (CST) on optical coherence tomography (OCT) [16].

In this study we report a real-world analysis of the effect of switching to faricimab in the treatment of nAMD, in terms of changes in treatment interval, best corrected visual acuity (BCVA), and fluid volumes as computed using machine learning (ML) analysis of OCT scans.

## Methods

### Study participants

This retrospective observational study examined data from all patients undergoing anti-VEGF therapy for the nAMD in the practices of eight retina specialists at five clinics in three Australian states (CORRNet Study Group; Clinical Ophthalmologists’ Real-world Research Network) between 2022–07-01 to 2024–07-11.

An Institutional Review Board (IRB) established that, given that there was no identifiable patient information collected in the study, it was exempt from IRB oversight. Written consent was obtained from all participants included in the study, allowing for the retrospective use of their clinical data. All patients had a clinical diagnosis of neovascular exudative AMD, based on clinical examination and macular imaging using criteria as previously reported [17].

Specifically, this retrospective study pertains to the subset of eyes in which the initial anti-VEGF agent(s) administered was switched to, and remained on, faricimab within the analysis period between 2023–01-01 to 2024–07-11, the former date being when faricimab was freely available for use in Australia. The data collected for each patient were limited to diagnosis for each eye, dates of all clinic visits, VA for each eye at every visit (using Snellen charts and transformed to LogMAR letters for analysis), treatment administered at each visit, and OCT scans for each visit. The OCT scans collected were either from the Cirrus OCT (Carl Zeiss Meditec; 6 × 6 mm, 512 × 128 macular cube, fovea-centered) or Spectralis OCT (Heidelberg Engineering; 20 × 20 degrees, 512 A-scans × 49- or 97-B-scans; fovea centered). Notably, all clinics used a single OCT device for all patients.

All eyes included in the study had both a minimum of six months’ history of previous treatment prior to and six months of follow-up after switch, with visual acuity measurements performed and OCT images acquired at every clinic visit. Data regarding the physicians'intention to treat and the reasons for switching were not collected, as these are inconsistently recorded and analyzing free text entries would be subjective.

Although this is a retrospective analysis, all retina specialists adhered to a TER [6] and did not have an overt tolerance for subretinal fluid [18]; all clinical decisions, including those to switch anti-VEGF agent, were at the sole discretion of the treating doctor, based solely on their clinical assessment. No machine learning algorithms for OCT analysis were available to them.

As this study is a retrospective real-world analysis, no exclusion criteria were established, other than data from patients that failed to attend follow up.

### OCT analysis

All OCT scans were analyzed for both the presence and volume of IRF and SRF using ML algorithms (Macuject Pty Ltd, Melbourne, Australia) trained and validated with separate datasets of images from those analysed [19]. Agreement between the algorithm and clinician grading was confirmed on a random subset of 30 eyes from this study cohort, showing a high correlation (Pearson r = 0.92 for SRF, r = 0.89 for IRF). Volumetric measurements were derived by integration and interpolation of fluid areas from each OCT B-scan in a cube, calibrated by using scan dimensions published by the OCT device manufacturer. The mean volume of each fluid compartment was calculated at each clinic visit. If a calculated volume measurement (either IRF, SRF or both) was 1nL or less, then the volume was deemed to be zero, i.e., fluid-free.

### Interval data analysis

For each clinic visit, the BCVA was defined as the highest recorded VA for each eye, whether it was unaided, with pinhole or with refraction. The treatment interval was defined, for each anti-VEGF injection, as the time elapsed (in weeks) since the previous anti-VEGF injection for that eye. The longest treatment interval for an eye at which the OCT scan was deemed to be fluid-free was designated the maximum fluid-free interval (FFI). This was then calculated for all treated eyes, by anti-VEGF agent and fluid compartment; IRF, whether alone or in combination with SRF, SRF whether alone or in combination with IRF, and for IRF and SRF when both were present.

### Statistical analysis

Statistical analysis was performed using the Statistical Package for the Social Sciences (SPSS Inc., version 20.0, Chicago, IL). Descriptive statistics were reported as mean and standard deviation for continuous variables or frequency and percentage for qualitative variables. Normality of the data was assessed using the Shapiro–Wilk test for all variables. Differences between all the variable values at the time of switch and after the switch were explored by using the Mann Whitney U test. The chosen level of statistical significance was set at *P* < 0.05.

## Results

### Fluid analysis

Of the 3082 eyes of 2200 patients treated with intravitreal anti-VEGF injections for nAMD, 779 eyes (25.27%) were switched to faricimab during the study period, with both six months or more preceding history and follow-up. Among these, 625 eyes (80.3%) continued their treatment with faricimab, and 153 (19.7%) were switched back to other drugs. At the time of switch, 563 eyes had ML analysis available for all prior clinic visits. Of the 563 eyes with ML analysis that were switched to faricimab, 451 (80%) were switched from aflibercept 2 mg, 99 (18%) were switched from ranibizumab and 13 (2.3%) from brolucizumab; none were switched from bevacizumab.

Of these, 473 eyes (84%) continued with, and ended, their therapy on faricimab. Thus, these eyes alone constituted the final analysis cohort for the present study; the 90 eyes (16%) that were subsequently switched away from faricimab to another anti-VEGF agent (76 eyes {84%} back to aflibercept 2 mg) are not included in these analyses.

The mean age of the final analysis cohort was 78.47 ± 5.29, and 52.4% of patients were female. The mean number of injections prior to initiating faricimab was 6.91 ± 2.8. The mean number of injections after the switch was 6.85 ± 4.2. The mean follow-up time after the switch was 10.1 ± 4.65 months. The characteristics of the eyes included in the analysis are reported in Table 1.

**Table 1:** Demographic characteristics of patients included in the analysis

Group	Number (%)
Age	78.47 ± 5.29
Gender *n*, (%)	225 (47.56%)
Mean follow-up after the switch	10.1 ± 4.65
Mean number of injections before the switch	6.91 ± 2.8
Mean number of injections after the switch	6.85 ± 4.2
Eyes	
• Total number	3082
• Switched to faricimab	779 (25.27%)
• Eyes with ML fluid analysis	563 (18.26%)
• ML fluid analysis and end with faricimab	473 (15.34%)
Drug from which switch to faricimab occurred	
• Aflibercept 2 mg	625 (80.2%)
• Brolucizumab	18 (2.3%)
• Ranimizumab	136 (17.5%)
Switched eyes remaining on faricimab	625 (80.3%)
Fluid subtype at the time of switch	
• SRF only	154 (32.6%)
• IRF only	73 (15.4%)
• Both IRF & SRF	69 (14.6%)
• Total number of eyes with fluid	296 (62,6%)
• No fluid	177 (37.4%)
Fluid subtype at the time of switch (whether in isolation or in combination)	
• SRF	223 (47.15%)
• IRF	142 (30.02%)
• Both	69 (14.59%)

At the time of switch to faricimab, 154 eyes had SRF only (32.6%), 73 had IRF only (15.4%), 69 (14.6%) had both fluid subtypes present, and 177 eyes (37.4%) had no fluid at the time of switch (Table 1). The number of eyes having IRF, either alone or in combination with SRF, was 142 (30.02%) and the number of eyes with SRF, either alone or in combination with IRF, was 223 (47.15%) (Table 1).

Following the switch to faricimab, for the 142 eyes with any IRF, 115 eyes (80.9%) had a reduction in IRF volume; of the 223 eyes with SRF, 190 eyes (85.2%) had a reduction in SRF volume, and 64 of the 69 (92.8%) eyes with both IRF and SRF present had a reduction in both fluid subtypes.

For all eyes included in the study, just before the first faricimab injection, the mean SRF volume was 39.2 ± 105.8 (SD) nL; the mean IRF volume was 9.07 ± 28.9 (SD) nL. The mean SRF volume after the switch was 19.26 ± 54.84 nL (*p* = 0.007), and mean IRF after the switch was 6.73 ± 21.13 nL (*p* < 0.001). Analyzing only the eyes having fluid at the time of switch, the mean SRF volume was 61.86 ± 127.5 (SD) nL and the mean IRF volume was 14.31 ± 35.2 (SD) nL. The mean SRF volume after the switch was 19.26 ± 54.84 nL (*p* = 0.007), and mean IRF after the switch was 6.73 ± 21.13 nL (*p* < 0.001). All these data referring to fluid volumes following switch are from the last recorded follow-up visit in the study.

Complete resolution of fluid, by subtype, occurred in 59 of the 142 eyes with IRF (41.6%), 81 of the 223 eyes with SRF (36.3%) and 16 of 69 (23.2%) eyes with both SRF and IRF.

For those eyes that had complete fluid resolution for either subtype or in combination, the time required following switch for the resolution to occur was a mean of 5.94 ± 2.69 (SD) weeks for IRF, 6.77 ± 4.75 (SD) weeks for SRF, and 6.51 ± 3.7 (SD) for IRF and SRF, when both fluid subtypes were present.

### Treatment interval analysis

At the time of switching, and thus relevant to the previous anti-VEGF agents, the maximum historical fluid free intervals for IRF, SRF and IRF + SRF were 6.41 ± 3.42 (SD), 5.49 ± 3.83 (SD), and 4.63 ± 3.88 (SD) weeks, respectively.

There was an overall increase in mean maximum fluid-free interval by the end of the study period. The magnitude of this was by 0.4 ± 4.09 weeks for IRF (*p* < 0.001), 0.79 ± 4.61 weeks for SRF (*p* < 0.001), and 0.87 ± 4.39 for both fluid subtypes (*p* < 0.01).

**Fig. 1:**
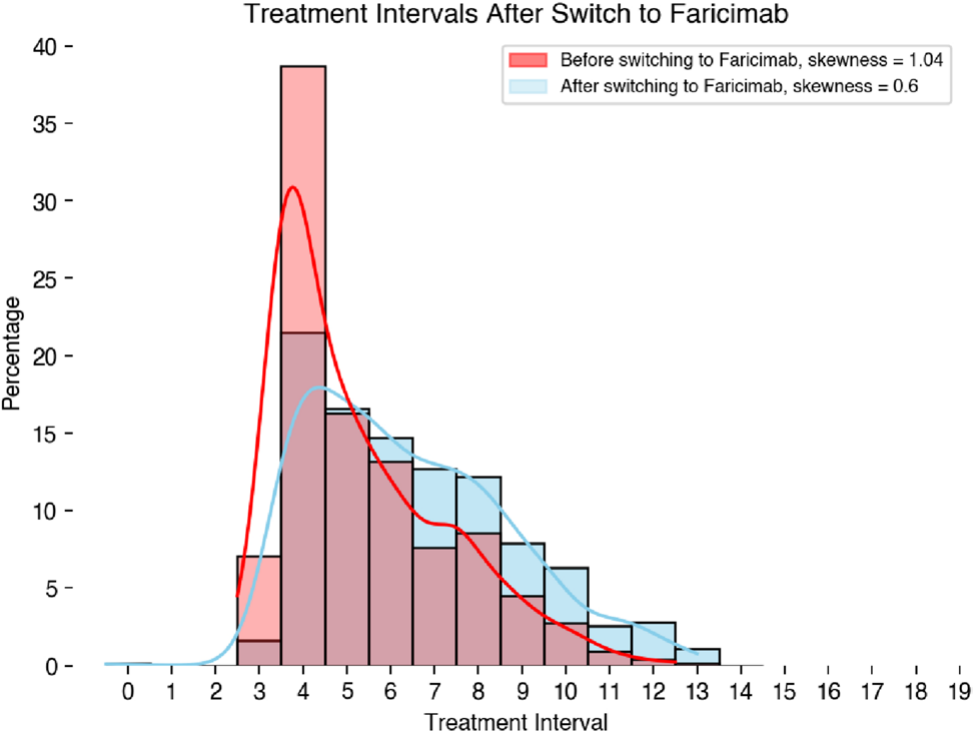
Difference between the intervals before and after the switch. The interval distribution shifted from a mean of 5.78 ± 1.92 to 6.91 ± 2.26 weeks over the study period (*p* < 0.001)

Irrespective of the fluid status, the interval distribution shifted from a mean of 5.78 ± 1.92 (SD) to 6.91 ± 2.26 (SD) weeks over the study period (*p* < 0.001) (Fig. 1).

### Visual acuity analysis

The mean best corrected visual acuity (BCVA) at the time of switch in eyes switched to faricimab was 63.91 ± 20.03 logMAR letters, improving to 69.25 ± 17.38 logMAR letters (*p* < 0.001) at the last recorded study visit.

## Discussion

Registration trials have already reported the efficacy and safety of faricimab in terms of VA recovery and fluid resolution in patients with nAMD [10]. Our real-world study focused on a “hard-to-treat” group of patients, most either with fluid at the time of switch despite an extensive period of previous anti-VEGF treatments, or at relatively short treatment intervals. We report a ML-based volumetric analysis of fluid, along with intervals and functional changes, in a group of patients switched to faricimab from other anti-VEGF drugs and remained on it. We do not, however, report on those that were subsequently switched back to another drug, which should be borne in mind when interpreting these data. Whilst it is likely that the eyes that were switched away from faricimab had poorer outcomes, either for fluid control or interval extension, these data are not available, as the reasoning behind physicians’ treatment decision-making was not collected.

Previous studies have reported the efficacy of faricimab in improving anatomical results after the switch from other drugs. These included one reporting a reduction of central subfield thickness (CST) of 38.1 μM after three injections [13]; another study, of 55 eyes resistant to previous treatments, found that a total of 39.3% of their patients achieved a CST of < 300 µm without retinal fluid after three injections of faricimab [14], and yet another reported a mean CST decrease of 20 µm after three injections of faricimab in patients resistant to previous intensive treatment with other drugs [15]. Recently, Han and coworkers showed the anatomical response during faricimab loading injections may vary depending on the subtype of MNV [20]. Unfortunately, given the insufficient clinical records, we were not able to analyze this point in the present study.

In our analysis we performed automated SRF and IRF volumetric qualification over the whole OCT cube/volume for every eye. CST is commonly used as a parameter of disease activity in nAMD, demonstrating anatomical improvement after intravitreal treatments. There is, moreover, no strong correlation with VA, even though there is evidence that IRF and SRF presence or absence are correlated with visual outcomes [16]. We would argue that automated ML segmentation and measurement of fluid compartment volumes is an absolute necessity for these relationships to be readily assessed. However, manual measurement is simply not a practical option outside of expensive industry-sponsored clinical trials.

Given the difficulty in comparing simple single dimension thickness measurements with three-dimensional volumetric data, it is helpful to consider these findings in the context of the measured differences in manual volumetric measurements between OCT scanners being within 10 nanoliters of each other [21]. Thus, the reductions in fluid volume, more so for SRF than IRF, were likely to be of clinical significance, although that has yet to be demonstrated empirically.

With respect to treatment intervals, following the switch, we observed a significant improvement, with a shift from a mean of 5.78 ± 1.92 (SD) to a mean of 6.91 ± 2.26 weeks over the study period (*p* < 0.001). Notably, all patients were managed with a T&E protocol, with no declared fluid tolerance. These results are in line with, but not as dramatic as, those observed by Sim and coworkers, which reported a mean extension of the intervals from 4.2 ± 0.3 to 6.9 ± 2.3 [15]. Interestingly, as with fluid compartment volumetrics, there seemed to be a differential effect on IRF and SRF compartments. Nevertheless, our results further confirm the ability of faricimab to reduce the treatment burden even in a “harder-to-treat” group of patients such as those included in our analysis. Importantly, though, the increase in fluid-free interval for each type of fluid was significantly less than a week; the overall increase in interval for the cohort examined must, therefore, include eyes that had fluid resolution, those in which there was no fluid at switch and cases where treatment interval extension occurred when fluid was present, whether noted or not by the treating physician.

Unlike previous reports [13, 15], however, we found a significant increase in BCVA after the switch. Indeed, we observed a mean BCVA improvement from 63.91 ± 20.03 logMAR letters at baseline, to 69.25 ± 17.38 logMAR letters (*p* < 0.001) after the switch. The latter results may be due to several factors including the timing of the switch. Indeed, it should be noted that a group of patients with a long history of previous anti-VEGF treatments, may develop several complications, such as macular atrophy or fibrosis, significantly influencing the functional outcomes.

Our study has several limitations, related to its real-world retrospective nature. A key component of this is the variability that is likely present both between and within the practices of the retina specialists involved. Although the general protocol adopted was TER, with no declared fluid tolerance, clinician- and patient-related factors may well have influenced decision-making, including which patients were switched. The present study collected no data on the physicians’ intention to treat and decision-making reasoning, along with an absence of safety data, and whether patients underwent cataract surgery following switch, potentially explaining the visual acuity improvement. Another limitation is that the subtype of MNV was not consistently documented in the clinical records and therefore could not be analyzed or reported in this study. Indeed, recent evidence suggests that the anatomical response during faricimab loading may vary by MNV subtype [20]. Given the purpose of this study to analyze fluid changes for each retinal compartement we aknoldge the importance of such limitation.

Another limitation of this study is the lack of analysis of fluid dynamics during the initial phase of treatment, specifically after one to three injections. We acknowledge that excluding this early-phase analysis may not fully capture the fluid response trajectory at the start of treatment. It is possible that in some cases, IRF and SRF may have resolved with further aflibercept injections, and the absence of this data may limit the interpretation of treatment resistance in the early course.

Importantly, it is unlikely that reimbursement or remuneration have played a role; in the Australian healthcare system all approved drugs are freely available and there is no financial consideration in the treatment choice, either for patients, doctors or payors. The absence of any patients switched from bevacizumab to faricimab is in striking contrast with US clinical practice. It is also important to specify that the results of the study cannot be directly compared to those of randomized clinical trials, as this is a real-world analysis with potential biases. In fact, the objective of our study is solely to report morpho-functional and volumetric changes in a population of patients switched to, and remaining on, faricimab rather than to speculate on the drug’s efficacy or indications for use. Furthermore, the absence of data on those patients switched back to another anti-VEGF agent means that overall conclusions cannot be drawn for all patients switched to faricimab, but solely to those who remained on faricimab. Therefore, these findings should be interpreted with caution given the retrospective nature of the study and the absence of a matched control group or comparative data from the pre-switch period, as well as the lack of distinction between prior treatments (i.e., aflibercept vs ranibizumab). Further prospective studies are warranted to more definitively evaluate the clinical benefits of faricimab in this setting.

A significant strength of our analysis is that it is one of the first large-scale real-world analyses assessing volumetric changes of fluid, treatment intervals, and VA changes, in a group of “hard-to-treat” nAMD eyes switched to faricimab. Improvements were seen in all three sets of treatment outcomes. Further subset analyses are warranted, especially with larger, prospective ML-assisted long-term real-world studies, both to corroborate these results and enable further structure–function and outcomes analyses. Such studies would also ideally collect and analyze data on eyes that were not switched to faricimab and those that switched back, for comparison. While switching to faricimab was associated with improved anatomical and functional outcomes in a majority of included cases, the exclusion of patients who required a subsequent switch to another agent limits the generalizability of our findings. Further studies are needed to confirm these results across a broader patient population.
